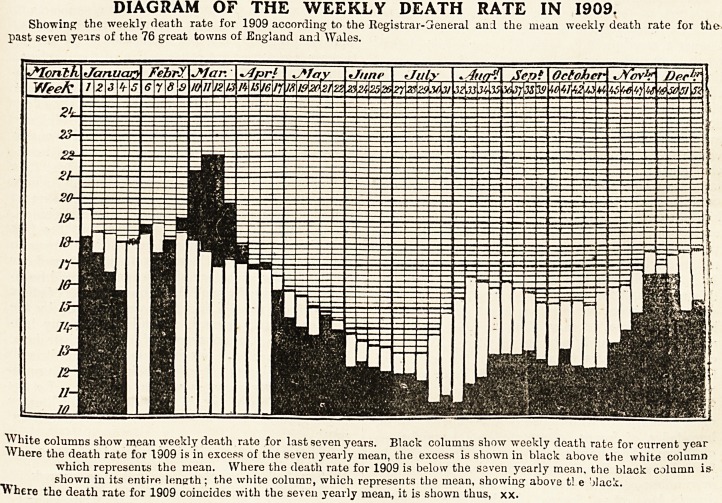# A Year of Low Mortality

**Published:** 1910-02-26

**Authors:** 


### Public Health and Hygiene.

### A YEAR OF LOW MORTALITY.

We have repeatedly commented on the succession of years recently experienced characterised by a low mortality. We have expressed the view that the condition most operative in producing this result is the phenomenal type of weather which has been associated with the more fatal seasons of these years. There has, in addition, been the accident of freedom from any epidemic presumably not dependent upon meteorological conditions. To insist unduly on the part played by purposive efforts to reduce the death-rate might be considered unwarranted assumption, but he would be sceptical indeed who denied that their rôle has been considerable. A study of the particular causes of death which in recent years have shown a marked decline reveals at once a coincidence with those to which most of all specific efforts have been directed to affect reduction. Most notable are infantile mortality and tuberculosis.

The increasing faith in the preventability of disease, the growing practice of the hygienic life, the spread of knowledge, and the more intimate community of medicine with the common life, are factors not easily assessed in the influence they have exerted; yet none will question their pervasive and profound effect upon the health and life of the people. We may or may not be reaping the harvest which the intrusion of medicine into social and domestic life has promised; the diagrams which depict so satisfactory an improvement in urban mortality may tell a darker story in the future, but we shall know that the application of knowledge and scientific control to the destructive forces of disease have not left unaltered those mortality curves which provoke, as the case may be, congratulation or dismay.

The urban mortality has in recent years been undergoing a phenomenal reduction. Influenza is one of those diseases which has so far failed to be influenced by the efforts of the sanitarian, and it has left its mark in the mortality curves of 1909. We have grown accustomed to the recurring epidemics of this uncontrolled zymotic. For the most part it is a mild and transient affection, and on this account provokes an unwarranted contempt which is responsible in large measure for its sharp tribute to mortality. A keener appreciation of the subtle dangers which attend its neglect, a deeper respect for the serious consequences which follow, especially among the aged and enfeebled, when the rules of the game it imposes are ignored, are the surest weapons with which at present it can be combated. It is to be feared, however, that while these remain the principal instruments in the armoury of preventive medicine, influenza will continue to leave an impress on mortality curves such as is illustrated in the diagram below in the March peak.

### DIAGRAM OF THE WEEKLY DEATH RATE IN 1909.

Showing the weekly death rate for 1909 according to the Registrar-General and the mean weekly death rate for the past seven years of the 76 great towns of England and Wales.

White columns show mean weekly death rate for last seven years. Black columns show weekly death rate for current year. Where the death rate for 1909 is in excess of the seven yearly mean, the excess is shown in black above the white column which represents the mean. Where the death rate for 1909 is below the seven yearly mean, the black column is shown in its entire length; the white column, which represents the mean, showing above the black.
Where the death rate for 1909 coincides with the seven yearly mean, it is shown thus, xx.

## Figures and Tables

**Figure f1:**